# Notch Signaling is Associated with ALDH Activity and an Aggressive Metastatic Phenotype in Murine Osteosarcoma Cells

**DOI:** 10.3389/fonc.2013.00143

**Published:** 2013-06-11

**Authors:** Xiaodong Mu, Christian Isaac, Nicholas Greco, Johnny Huard, Kurt Weiss

**Affiliations:** ^1^Stem Cell Research Laboratory, University of Pittsburgh Medical Center, Pittsburgh, PA, USA; ^2^Department of Orthopaedic Surgery, University of Pittsburgh School of Medicine, Pittsburgh, PA, USA

**Keywords:** Notch, ALDH, osteosarcoma, metastasis, mouse osteosarcoma cells

## Abstract

Osteosarcoma (OS) is the most common primary malignancy of bone, and pulmonary metastatic disease accounts for nearly all mortality. However, little is known about the biochemical signaling alterations that drive the progression of metastatic disease. Two murine OS cell populations, K7M2 and K12, are clonally related but differ significantly in their metastatic phenotypes and therefore represent excellent tools for studying metastatic OS molecular biology. K7M2 cells are highly metastatic, whereas K12 cells display limited metastatic potential. Here we report that the expression of Notch genes (Notch1, 2, 4) are up-regulated, including downstream targets Hes1 and Stat3, in the highly metastatic K7M2 cells compared to the less metastatic K12 cells, indicating that the Notch signaling pathway is more active in K7M2 cells. We have previously described that K7M2 cells exhibit higher levels of aldehyde dehydrogenase (ALDH) activity. Here we report that K7M2 cell ALDH activity is reduced with Notch inhibition, suggesting that ALDH activity may be regulated in part by the Notch pathway. Notch signaling is also associated with increased resistance to oxidative stress, migration, invasion, and VEGF expression *in vitro*. However, Notch inhibition did not significantly alter K7M2 cell proliferation. In conclusion, we provide evidence that Notch signaling is associated with ALDH activity and increased metastatic behavior in OS cells. Both Notch and ALDH are putative molecular targets for the treatment and prevention of OS metastasis.

## Introduction

Osteosarcoma (OS), the most common primary malignancy of bone, usually occurs in the long bones of youths in the first and second decades of life (Bielack et al., 2002; Carrle and Bielack, 2006; Hayden and Hoang, 2006). Despite pre- and post-operative chemotherapy with wide surgical resection of the tumor, overall survival for patients with OS is only 65–70% (Bielack et al., 2002; Ferrari et al., 2005; Carrle and Bielack, 2006; Meyers et al., 2008; Messerschmitt et al., 2009). The presence of metastatic disease at the time of diagnosis is clinically detectable in one out of every five cases, and portends a particularly poor prognosis (Bielack et al., 2002; Meyers et al., 2005, 2008). Therefore, the presence of pulmonary metastases is typically responsible for OS mortality (Meyers and Gorlick, 1997). However, little is known about the biochemical signaling pathways that are critical for the progression of metastatic disease, and the molecular biology of OS remains poorly understood. As a result, we have yet to develop any efficacious drugs that specifically target metastatic disease.

Notch signaling is critical for many cellular processes including development and tissue homeostasis. For this reason, Notch has been called “the stem cell master switch” (Artavanis-Tsakonas et al., 1999; Lai, 2004). Its role in the formation and progression of cancer has also been reported (Bray, 2006). Although a role for Notch signaling in cancer metastasis is evolving, our current understanding is limited (Hu et al., 2012). The Notch family of transmembrane receptors is activated when their counterpart transmembrane ligands bind to the extracellular domain of Notch. This receptor-ligand interaction is mediated by cell–cell contacts and initiates the cleavage of Notch intracellular domain (NICD) by the Presenilin family of gamma-secretases. NICD is then free to translocate to the nucleus and initiate a variety of genetic programs through the regulation of gene transcription. Notch signaling is vastly complex and its influence on cellular processes is highly dependent on cellular context, lineage, commitment, and timing (Bray, 2006). Notch signaling plays a critical role in cell proliferation, differentiation, and apoptosis. Notch dysregulation is felt to be responsible for a variety of human cancers including leukemia, pancreas, breast, prostate, melanoma, and gastrointestinal tract neoplasia (Miyamoto et al., 2003; Weng et al., 2004; van Es et al., 2005; Bray, 2006; Wang et al., 2010; Hu et al., 2012). In addition, it was recently discovered that Notch signaling is activated in human OS and may play a role in tumor invasion and metastasis (Engin et al., 2008; Zhang et al., 2008, 2010). The primary goal of this study was to investigate the role of Notch signaling in OS metastasis.

K7M2 murine OS cells are highly metastatic to the lungs and were clonally derived from the much less metastatic K12 OS cells (Khanna et al., 2000). The K7M2 and K12 cell populations are related but differ dramatically in their metastatic phenotypes. They therefore represent excellent tools for determining the critical biochemical pathways in OS metastasis. After comparing these cell lines *in vitro*, we report that Notch signaling is up-regulated and associated with higher aldehyde dehydrogenase 1A1 activity aldehyde dehydrogenase (ALDH, a cancer stem cell marker) (Marcato et al., 2011), increased resistance to oxidative stress, migration, invasion, and VEGF expression, but is not essential for the proliferation of K7M2 and K12 cells, suggesting that alternative or compensatory signal transduction pathways may play a role in OS cell proliferation in this system. These experiments provide evidence that the Notch pathway is critical for OS metastasis and may regulate ALDH activity. Notch is thus further implicated as a putative target in the treatment and prevention of OS metastasis.

## Materials and Methods

### Cell culture and DAPT treatment

K7M2 cells and K12 cells were cultured with proliferation medium (PM, DMEM with 10% FBS and 5% penicillin and streptomycin). For Notch inhibition of K7M2 cells, the Notch gamma-secretase inhibitor DAPT (*N*-[*N*-(3,5-difluorophenacetyl)-l-alanyl]-s-phenylglycine *t*-butyl ester, from Sigma) was dissolved in DMSO (10 mM), and diluted 1:1000 in PM to a working concentration of 10 μM. K7M2 cells were seeded in 12-well plastic plates at 5000 cells per well, and 1 mL treatment medium containing DAPT was added each well. One microliter medium containing same amount of DMSO without DAPT served as control treatment. Treatment medium was refreshed each day and cells were treated for 2–4 days.

### Notch activation in K12 cells with Jagged1

K12 cells were treated with active Jagged1 peptide (CDDYYYGFGCNKFCRPR) or scrambled peptide (RCGPDCFDNYGRYKYCF) (GenScript USA, Inc.; 20 μg/mL) in PM for 4 days to compare their various properties. Treatment medium was refreshed each day. Fluorescence-activated cell sorting (*FACS*) *Analysis of ALDH Activity*. The Alde lfluor Kit (STEMCELL Technologies) was used in this analysis. Cultured K7M2 cells and K12 cells, with or without DAPT treatment (10 μM for 48 h) were resuspended in Aldefluor buffer (1 × 10^6^ cells/mL), and incubated at 37° C according to the manufacturer’s instructions. Cells were washed in Aldefluor buffer and maintained in 4° C throughout the analysis process. ALDH activity was assessed using the FL1 channel of a BD FACSAria Cell Sorting System and FACSDiva software (version 6.1.2; Becton, Dickinson and Company, San Jose, CA, USA). Collected cells were gated on their fluorescence intensity, which corresponds to their ALDH activity levels, as well as low side scatter (SCC^*lo*^).

### Fluorescence-activated cell sorting by ALDH activity

Cultured K7M2 cells were trypsinized, washed in cold PBS, and counted using a hemocytometer. Cells (10^6^) of each population were resuspended in Aldefluor buffer, which contains an ABC transport inhibitor that prevents efflux of the Aldefluor dye, and incubated at 37° C according to the manufacturer’s instructions. Cells were washed in Aldefluor buffer and maintained in 4° C throughout the cell sorting process. Collected cells were gated on their fluorescence intensity, which corresponds to their ALDH activity levels, as well as low side scatter (SCC^*lo*^). Sorted cells were recaptured in cold (4° C) PM and immediately plated in collagen-I coated flasks and normal incubation conditions (5% CO_2_ at 37° C).

### Cell proliferation assay

K7M2 and K12 cells were plated at 1000 cells per well in 12-well plates and cultured in PM. A time-lapsed microscopic live-cell imaging (LCI) system (Automated Cell, Inc.) was used to take images of cells per field of view at 15 min intervals for 4 days. The approximate population doubling time (PDT) as determined as follows: 2*^*n*^* = cell number at harvest time/cell number initially plated; “*n*” refers to the number of doublings during the period of cell culture (96 h), thus PDT = 96 h/*n*.

### Cell survival assay after exposure to oxidative stress

To test the role of Notch inactivation on the antioxidant capacity in K7M2 cells, K7M2 were pre-treated with DAPT (10 μM) for 48 h prior to exposure to oxidative stress (0, 250, or 500 μM H_2_O_2_ in PM) conditions for 6 h. Propidium iodide (PI) was added to the medium (1 μg/mL) and apoptotic cells were identified with positive PI staining.

### Cell migration study with *in vitro* wound repair assay

K7M2 cells with or without treatment with DAPT (10 μM for 48 h) were grown to near confluence in a 12-well multi-well plate. Artificial wounds were created by disrupting cell monolayers with a sterile pipette-tip. Cellular debris was aspirated and fresh PM was added to the wells. Images of cell migration into the artificial wound were taken at 0 and 9 h after creating the artificial wound as previously described (Mu et al., 2010). Cell migration was measured in microns (μm) by the distance traveled into wound site.

### Cell migration study with *in vitro* invasion assay

*In vitro* invasion capacity of K7M2 and K12 cells was assessed using a real-time cell invasion and migration (RT-CIM) assay system (ACEA Biosciences, Inc.), with a 16-well trans-well plate (CIM-plate 16, Roche Diagnostics GmbH). The surface of the wells in the upper chamber was coated with Matrigel (BD BioSciences, Bedford, MA, USA) of different concentrations (2.5, 5, and 10%). Serum-containing medium (10% FBS) was added to the wells of the lower chamber. Cells (4 × 10^4^ per well) in serum-free medium were seeded in the upper chamber. The migration of the cells through the Matrigel was monitored by the system every 15 min for 18 h. Data analysis was carried out using RTCA Software 1.2 supplied with the instrument.

### Reverse transcription-PCR

Total RNA was extracted from the cells using the RNeasy plus mini kit (Qiagen) and cDNA was generated using the iScript cDNA Synthesis kit (Bio-Rad). The sense and anti-sense primers for reverse transcription polymerase chain reaction (RT-PCR) and their product sizes are found in the Table 1. The cycling parameters used for all reactions were as follows: 94° C for 5 min; 30 cycles of the following: denature for 45 s at 95° C, anneal for 30 s (53–56° C), and extend for 45 s at 72° C. RT-PCR was performed using a Bio-Rad MyiQ thermal cycler (Bio-Rad).

**Table 1:** **Primer sequences**.

Gene	Primer sequence
GAPDH	Forward: TCCATGACAACTTTGGCATTG
	Reverse: TCACGCCACAGCTTTCCA
Notch1	Forward: GCCGCAAGAGGCTTGAGAT
	Reverse: GGAGTCCTGGCATCGTTGG
Notch2	Forward: GAGAAAAACCGCTGTCAGAATGG
	Reverse: GGTGGAGTATTGGCAGTCCTC
Notch3	Forward: TGCCAGAGTTCAGTGGTGG
	Reverse: CACAGGCAAATCGGCCATC
Notch4	Forward: CCAGAATGCGAGACAGAACTG
	Reverse: GGTCAACCCCATGTAGCCTG
Hes1	Forward: CCAGCCAGTGTCAACACGA
	Reverse: AATGCCGGGAGCTATCTTTCT
Stat3	Forward: CAATACCATTGACCTGCCGAT
	Reverse: GAGCGACTCAAACTGCCCT
c-Myc	Forward: TGACCTAACTCGAGGAGGAGCTGGAATC
	Reverse: AAGTTTGAGGCAGTTAAAATTATGGCTGAAGC
BMP2	Forward: TCTTCCGGGAACAGATACAGG
	Reverse: TGGTGTCCAATAGTCTGGTCA
BMP4	Forward: ATTCCTGGTAACCGAATGCTG
	Reverse: CCGGTCTCAGGTATCAAACTAGC
VEGF	Forward: GCCAGACAGGGTTGCCATAC
	Reverse: GGAGTGGGATGGATGATGTCAG
ALDH	Forward: GACAGGCTTTCCAGATTGGCTC
	Reverse: AAGACTTTCCCACCATTGAGTGC

### Measurement of results and statistical analysis

Reverse transcription-polymerase chain reaction analysis was performed using ImageJ software (version 1.32j, National Institutes of Health, Bethesda, MD, USA) where the integrated density (product of the area and the mean gray value) of bands was calculated. All molecular bands were represented as a percentage of a standard gene, GAPDH. At least three samples obtained from each subject were pooled for statistical analysis of all results from this study, and the results are expressed as a mean ± SD. The differences between two means were considered to be statistically significant if *P* value is<0.05. A student’s *t*-test was used to determine whether there were statistically significant differences between two means.

## Results

### Notch signaling is up-regulated in K7M2 cells

**Figure 1:**
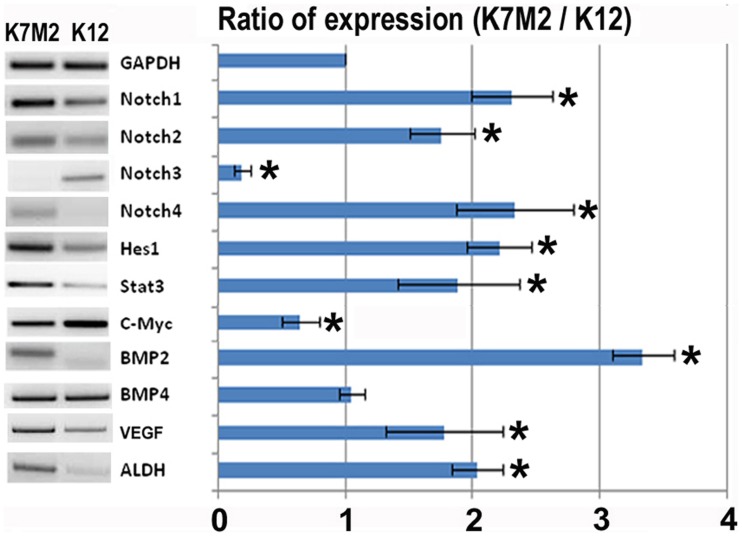
**Notch activation and ALDH activity in K7M2 cells**. RT-PCR was performed on cellular RNA extracted from K7M2 and K12 cells in order to quantitate the relative expression of Notch genes 1, 2, 3, 4, and canonical downstream targets of notch, Hes1, STAT3, and C-myc. In addition, ALDH gene expression was compared between K7M2 and K12 cells. BMP2 and BMP4 were included as positive and negative controls. GAPDH serves as a loading control for both lanes. The RT-PCR results were quantitated and the results are displayed in graph representation. Gene expression was normalized using GAPDH. The ratio of K7M2 gene expression to K12 gene expression is shown. *Indicates the difference is significant comparing DAPT treated or non-treated samples (*P* < 0.05).

In order to investigate the role of Notch signaling in OS metastasis we first performed RT-PCR to analyze the expression of Notch genes, and their canonical downstream target genes, Hes1 and Stat3, in K7M2 and K12 cells (Kamakura et al., 2004; Borggrefe and Oswald, 2009). Using this system we have previously shown that Bmp2 and VEGF expression are up-regulated in K7M2 cells compared to K12 cells (Weiss et al., 2006). Therefore, we included Bmp2 and VEGF as historical controls in addition to the loading control, GAPDH. We observed a significant up-regulation of almost twofold in each of Notch-1, -2, and -4 transcripts, with concomitant up-regulation of the Notch target genes, Hes1 and Stat3, in K7M2 cells compared to K12 cells (Figure 1). Interestingly, Notch3 expression was significantly down-regulated in K7M2 cells compared to K12 cells. Likewise, and somewhat surprisingly, c-Myc expression, a proto-oncogene known to be positively regulated by Notch signaling (Klinakis et al., 2006), was also significantly down-regulated in K7M2 cells.

Aldehyde dehydrogenase activity is found in a variety of human cancers and has been associated with metastasis, drug resistance, and poor prognosis (Cheung et al., 2007; Cho et al., 2007; Huang et al., 2009; Charafe-Jauffret et al., 2010; Honoki et al., 2010). Here we observed a twofold up-regulation of ALDH gene transcript levels in K7M2 cells (Figure 1). In our previous study we have performed FACS analysis to quantitate the percentage of cells with ALDH activity in K7M2 and K12 cells (Mu et al., 2012). Consistent with our RT-PCR results, we observed a threefold increase in the percentage of K7M2 cells with high ALDH activity compared to K12 cells.

**Figure 2:**
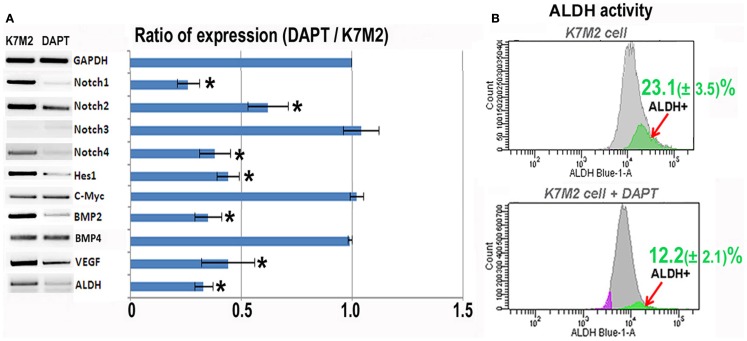
**Notch inhibition with DAPT reduces Notch signaling and BMP2, VEGF, and ALDH expression in K7M2 cells**. **(A)** RT-PCR was performed on cellular RNA extracted from K7M2 cells treated with DAPT or vehicle only (control) in order to quantitate the relative expression of Notch genes, and Notch targets Hes1 and C-myc. In addition, BMP2, VEGF, and ALDH gene expression was compared between DAPT-treated, and untreated, K7M2 cells. GAPDH serves as a loading control for both lanes. The RT-PCR results were quantitated and the results are displayed in graph representation. Gene expression was normalized using GAPDH. **(B)** ALDH activity was detected in DAPT-treated and untreated K7M2 cells using flow cytometric analysis and the relative amount of cells positive for ALDH is shown for each cell population. *Indicates the difference is significant comparing DAPT-treated or non-treated samples (*P* < 0.05).

In order to investigate the relationship between Notch activation and ALDH activity in the highly metastatic K7M2 cells we inhibited Notch signaling with the gamma-secretase inhibitor, DAPT (Geling et al., 2002). We then performed RT-PCR to confirm the inhibition of Notch activity. Figure 2A shows a significant reduction in Notch1, -2, -4, and Hes1 gene expression in K7M2 cells after culture in DAPT-conditioned medium, whereas, we did not observe a significant alteration in Notch3, c-Myc, or Bmp4 transcript levels after exposure to DAPT. Interestingly, the expression of ALDH was significantly down-regulated after DAPT treatment, as was Bmp2 and VEGF expression. In order to further investigate ALDH activity in the setting of Notch inhibition, we utilized FACS and observed a near twofold reduction in cells positive for ALDH activity (12.2 ± 2.1 vs. 23.1 ± 3.5%, *P* < 0.05) after treatment with DAPT (Figure 2B). After confirming that DAPT reduces Notch signaling, ALDH expression, and ALDH activity in K7M2 cells, we wanted to investigate the effects of DAPT treatment on K7M2 cell proliferation, resistance to oxidative stress, migration, and invasion *in vitro* as these are phenotypes that confer metastatic potential (Hu et al., 2012).

### Notch inhibition sensitizes K7M2 cells to oxidative stress but does not alter proliferation

**Figure 3:**
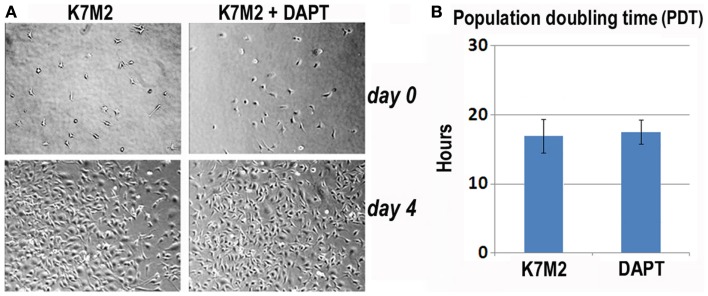
**Notch inhibition with DAPT does not affect proliferation of K7M2 cells**. **(A)** K7M2 cells were cultured with media containing 10 μM DAPT or vehicle only (control) for 4 days. Representative images of cell density at the beginning and end of treatment are shown. Similar number of cells was observed between the two groups. **(B)** The population doubling time of K7M2 cells with or without DAPT treatment showed no significant difference.

First, we analyzed K7M2 cell proliferation with and without DAPT treatment. After 4 days of culture, we did not observe a change in cell density as illustrated by the representative images in Figure 3A, and there was no significant change in cell population growth between K7M2 cells treated with or without DAPT (Figure 3B).

**Figure 4:**
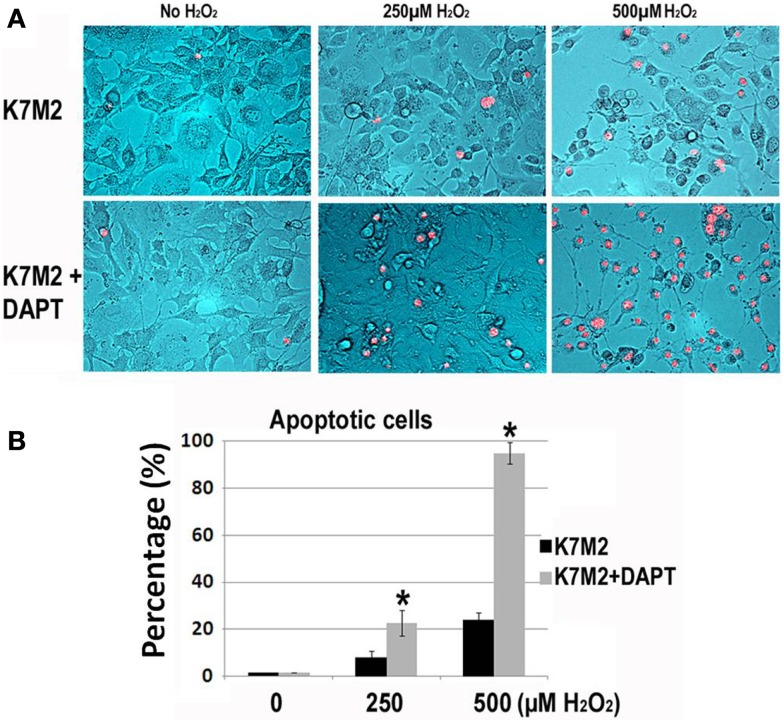
**K7M2 cells are resistant to treatment with H_2_O_2_ but become sensitive in the presence of DAPT**. **(A)** K7M2 cells were treated with or without DAPT (10 μM) for 4 days, and were then cultured with media containing H_2_O_2_ (0, 250, or 500 μM) for 6 h and cell death was analyzed using PI exclusion assay. Representative images are shown. **(B)** The percentage of PI+ cells was determined for each group in **(A)**. *Indicates the difference is significant comparing DAPT-treated or non-treated samples (*P* < 0.05).

Next, we tested resistance to oxidative stress using H_2_O_2_ treatment. Apoptosis was monitored by nuclear inclusion of PI. In our previous study we have shown that after treatment with H_2_O_2_, the large majority (>85%) of K12 cells underwent apoptosis as indicated by PI inclusion, whereas most of the K7M2 cells maintained viability (>65%) as indicated by nuclear exclusion of PI (Mu et al., 2012). Therefore, K7M2 cells are more resistant to oxidative stress from H_2_O_2_ exposure than K12 cells. In order to test if this resistance to H_2_O_2_ was related to Notch or ALDH activity we repeated these experiments on K7M2 cells in the presence or absence of DAPT and 0, 250, or 500 μM of H_2_O_2_. Representative images are shown in Figure 4. We observed a dose-dependent sensitivity to H_2_O_2_ with DAPT treatment as DAPT caused a twofold increase in apoptosis at 250 μM H_2_O_2_, and greater than a fourfold increase in apoptosis at 500 μM H_2_O_2_. DAPT alone is not pro-apoptotic in this assay, as DAPT in the absence of H_2_O_2_ did not increase the frequency of PI nuclear inclusion in K7M2 cells.

### Notch inhibition reduces K7M2 cell migration and invasion *in vitro*

**Figure 5:**
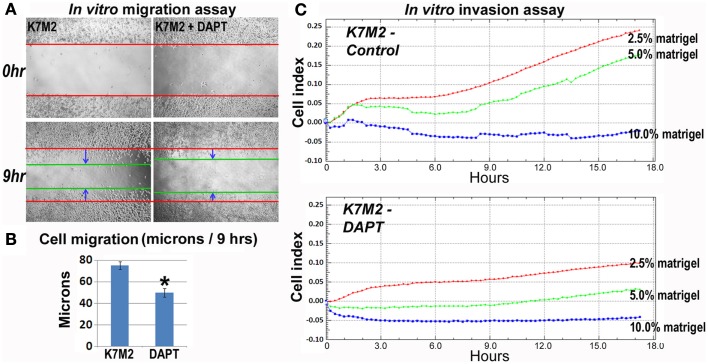
**K7M2 cells have greater migratory capacity and this is inhibited by DAPT treatment**. **(A)** Migratory ability of untreated K7M2 cells and DAPT-treated K7M2 cells was analyzed by cell migration study with *in vitro* wound repair assay, and representative images are shown. **(B)** The speed of migration was determined in each group in **(A)** and the results are displayed. **(C)** DAPT treatment resulted in decreased *in vitro* invasion capacity of K7M2 cells in 2.5, 5, or 10% matrigel. *Indicates the difference is significant comparing DAPT-treated or non-treated samples (*P* < 0.05).

As mentioned, K7M2 cells are much more metastatic than K12 cells (Khanna et al., 2000). Consistent with this enhanced metastatic potential, we performed *in vitro* migration and invasion assays in our previous study and observed K7M2 cells to migrate and invade significantly faster than K12 cells (Mu et al., 2012). Here we tested the effect of DAPT treatment on the ability of K7M2 cells to perform in these migration and invasion assays. Figure 5A is a representative image depicting the migration front of K7M2 cells after 9 h of treatment with and without DAPT. We observed a significant reduction in K7M2 cell migration distance after 9 h of DAPT treatment when compared to control (Figure 5B). Similarly, we observed a significant reduction in cell index achieved by K7M2 cells in an invasion assay through 2.5, 5, and 10% Matrigel after 18 h of DAPT treatment (Figure 5C). In our assays, K12 cells did not invade the 10% Matrigel effectively and this was unaffected by the presence of DAPT.

### Activation but not inactivation of Notch signaling in K12 cells increased the metastatic properties

**Figure 6:**
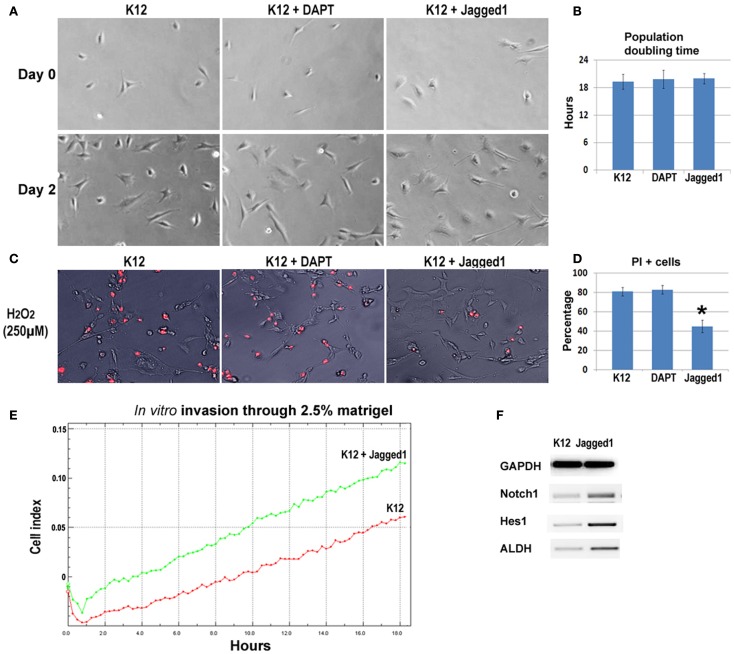
**Inhibition and activation of Notch signaling in K12 cells with DAPT and Jagged1**. **(A)** DAPT or Jagged1 treatment of K12 cells showed no effect on cell proliferation. **(B)** Statistics of population doubling time (PDT) with or without treatment. **(C)** Jagged1 treatment, but not DAPT treatment of K12 cells resulted in reduced cell apoptosis induced by H_2_O_2_ (250 μM; PI staining). **(D)** Statistics of PI+ cells with or without treatment. **(E)** Jagged1 treatment of K12 cells increased the *in vitro* invasion capacity of the cells through 2.5% matrigel. **(F)** RT-PCR showed that the expression of Notch1, Hes1, and ALDH1A1 was up-regulated in K12 cells with Jagged1 treatment.

To further verify the involvement of Notch signaling in regulating metastatic properties of OS cells, K12 cells expressing lower level of Notch genes were treated with DAPT and a Notch activating ligand, Jagged1. Results revealed that neither DAPT nor Jagged1 treatment had an obvious effect on the proliferation of K12 cells (Figures 6A,B), but Jagged1 treatment specifically increased the anti-oxidative stress capacity of the cells (Figures 6C,D). Also, the *in vitro* invasion capacity of K12 cells was increased by Jagged1 treatment for 4 days (Figure 6E). Moreover, the expression of Notch1, Hes1, and ALDH1A1 was found to be up-regulated with Jagged1 treatment of K12 cells (Figure 6F).

### Sorted ALDH-high and ALDH-low K7M2 cells feature differential Notch expression and anti-oxidative stress capacity

**Figure 7:**
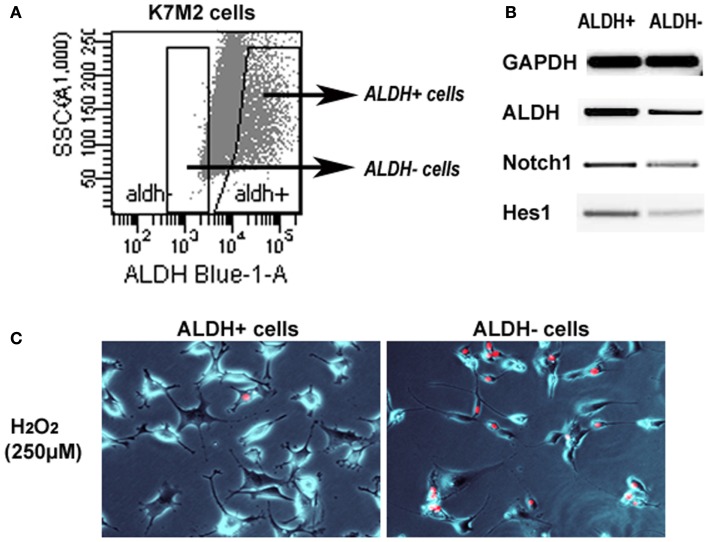
**Sorted ALDH-high and ALDH-low K7M2 cells show different expression patterns and resistance to oxidative stress**. **(A)** Demonstration of the strategy for sorting of ALDH-high and ALDH-low cells. **(B)** ALDH-high cells featured higher expression of Notch1, Hes1, and ALDH1A1, compared to ALDH-low cells. **(C)** ALDH-high cells demonstrated reduced cell apoptosis induced by H_2_O_2_ (250 μM; PI staining), compared to ALDH-low cells.

We have previously sorted K7M2 cells according to ALDH activity (Figure 7A) (Mu et al., 2012), and found ALDH-high cells demonstrated stronger *in vitro* invasion capacity than ALDH-low cells. Here, to further verify the involvement of Notch signaling in regulating ALDH activity, we have compared the expression of Notch genes in the two types of cells, and found up-regulated expression of Notch1, Hes1, and ALDH1A1 genes in ALDH-high K7M2 cells (Figure 7B). Also, the anti-oxidative stress capacity of ALDH-high cells was found much higher than that of ALDH-low cells (Figure 7C). These observations further implicated there is potential interaction between Notch signaling with ALDH in our system.

## Discussion

K7M2 murine OS cells are highly metastatic to the lungs and were clonally derived from the much less metastatic K12 OS cells (Khanna et al., 2000). Thus, the K7M2 and K12 cell lines are similar but differ drastically in their metastatic phenotypes, and therefore represent an excellent tool for determining critical biochemical pathways in OS metastasis. Here we report that the Notch genes (1, 2, and 4) are highly expressed, including downstream targets Hes1 and Stat3, indicating that the Notch signaling pathway is more active in K7M2 cells. These results imply that Notch signaling may contribute to the enhanced metastatic ability of K7M2 cells. To support this interpretation we have provided *in vitro* evidence illustrating that Notch activity is associated with many cellular behaviors important for metastasis. Notch inhibition via DAPT treatment also reduces ALDH activity and inhibits resistance to oxidative stress, cellular migration, invasion, and VEGF expression (a strong promoter of angiogenesis) (Witzenbichler et al., 1998; Zhang et al., 2000); all key cellular processes that required for metastatic progression (Hu et al., 2012).

A role for Notch signaling in OS metastasis is supported in the work published by Zhang et al. (2008) who found Notch1, Notch2, DLL1, and Hes1 over-expression in a number of human OS tumor samples and cell lines, and observed that compound E (a gamma-secretase inhibitor) reduced Matrigel invasion by these OS cells. The authors also implicated Notch signaling in OS metastasis by showing that expression of dominant negative Mastermind was able to block OS187 metastasis in a xenograft model. More recently the same group provided evidence that reciprocal inhibition of two downstream targets of Notch, Hes1, and Deltex1, may be the transcriptional control mechanisms that regulate cellular invasiveness in OS (Zhang et al., 2010).

Aldehyde dehydrogenase activity has been identified as a “cancer stem cell” (CSC) marker in numerous neoplasia. ALDH has been associated with metastasis and poor prognosis but such a role has yet to be established in OS (Cheung et al., 2007; Cho et al., 2007; Huang et al., 2009; Charafe-Jauffret et al., 2010; Honoki et al., 2010). The CSC hypothesis predicts that if certain genetic alterations occur in the right context, and within a more primitive cell, then a cancer initiating cell is born that retains all the qualities of stem cells including, self renewal, proliferation, differentiation, resistance to drugs, stress, and apoptosis, and perhaps most importantly, the ability to migrate, invade, and induce angiogenesis (Wicha et al., 2006). Although the relationship between CSCs and metastasis has not been clearly elucidated, it has been demonstrated that the number of metastatic cancer colonies correlates with the frequency of CSCs in the primary tissue. Furthermore, CSC subpopulations display a higher potential for invasiveness than subpopulations of non-stem tumor cells (Hu et al., 2012).

In terms of stem cell regulation, Notch is important for self renewal, proliferation, and maintenance of progenitor cells (Bray, 2006). Interestingly, altered Notch1 signaling alone in a T cell progenitor causes T cell acute lymphoblastic leukemia suggesting that Notch1 dysregulation may be a critical event for the CSC in this malignancy (Weng et al., 2004). Although an OS stem cell has not been identified to date, evidence suggests that this discovery is merely a matter of time (Gibbs et al., 2011). Notch signaling indeed has the potential to regulate an OS stem cell as recent genetic evidence links Notch activity to osteoblast differentiation, proliferation, and lineage commitment. This demonstrates that Notch plays a key role in osteoprogenitor cell maintenance and bone homeostasis (Engin et al., 2008; Hilton et al., 2008). If an OS stem cell exists, we suspect that both Notch and ALDH activity play pivotal roles in the maintenance of this subpopulation of cancer cells. ALDH activity may prove to be a useful marker to identify the cells within a tumor that have OS stem cell characteristics.

In this study, we observed that K7M2 cells exhibit higher levels of ALDH activity and this is dependent on Notch signaling as indicated by treatment with a gamma-secretase inhibitor. Furthermore, we showed that K7M2 cells, in comparison to K12 cells, are more resistant to oxidative stress induced by H_2_O_2_ treatment. Treating less metastatic K12 cells with the Notch activator Jagged1 increased the resistance of these cells to oxidative stress, enhanced their invasiveness, and altered their expression of Notch1, Hes1, and ALDH. It did not alter their proliferation. The activity of ALDH in cancer may function to neutralize oxidative stress and provide chemoresistance. Up-regulation of ALDH activity by Notch may be the mechanism that confers resistance to oxidative stress in K7M2 cells (Russo and Hilton, 1988; Magni et al., 1996). Alternatively, disulfiram, an ALDH inhibitor, has been shown to reduce the invasiveness of U2OS cells, and the expression of matrix metalloproteinases, raising the possibility that ALDH activity may have a more direct role in tumor invasion (Cho et al., 2007). We have shown recently that disulfiram reduces ALDH activity in K7M2 cells, and is associated with morphologic changes as well (Mu et al., 2012). However, the mechanism for disulfiram’s effect on OS cell invasion remains unclear.

It is somewhat surprising that DAPT did not significantly alter the proliferation of K7M2or K12 cells because Notch signaling is known to be pro-proliferative. We do believe this speaks to the complexity of Notch signaling and illustrates that the outcome of Notch activity depends on the cellular context and is probably influenced by many factors. In terms of the regulation of cellular proliferation, transcriptional activation of the proto-oncogene c-Myc by NICD can promote S-phase entry through the up-regulation of cyclins and the down-regulation of cyclin-dependent kinase inhibitors (Klinakis et al., 2006). The molecular result is the inactivation of the retinoblastoma protein (pRB) by phosphorylation which allows for transcription of E2F genes necessary for the cell cycle to advance through G1 (van den Heuvel and Dyson, 2008). In OS, pRB is frequently inactivated by direct mutation (Wang, 2005; Hayden and Hoang, 2006), and thus, c-Myc expression may not be necessary in this context for cell proliferation. This may explain why down-regulation of c-Myc in K7M2 cells does not cause growth arrest. Furthermore, inhibition of Notch signaling may have no effect on proliferation in K7M2 or K12 cells simply because pRB may be inactivated by another mechanism, and thus is essentially uncoupled from Notch activity.

## Conclusion

Notch signaling is important in many cancers and has been identified as both a tumor suppressor and an oncogene, illustrating the inherent complexity of this signaling pathway (Radtke and Raj, 2003; Weng et al., 2004; Bray, 2006). Our work supports an oncogenic role for Notch in OS and provides evidence to link Notch signaling, ALDH activity, and metastatic potential. To our knowledge the present study is the first to implicate Notch in the regulation of ALDH activity. Given our data and other evidence that implicates Notch and ALDH function in CSCs, we suspect that if an OS cancer stem cell (OSC) exists, Notch and ALDH may be important for OSC maintenance, drug resistance, and metastasis, and ALDH may be a potential OSC surface marker. At this point in time we cannot be certain that ALDH is downstream of Notch. These and other ideas regarding the relationships between Notch, ALDH, and the putative OSC require further investigation.

In conclusion, Notch signaling is up-regulated and associated with increased ALDH expression, ALDH activity, resistance to oxidative stress, migration, invasion, and VEGF expression *in vitro*. It is not essential for growth, suggesting that alternative or compensatory signal transduction pathways may play a role in OS cell proliferation. This work implicates Notch as a putative target in the treatment and prevention of OS metastasis.

## Conflict of Interest Statement

The coauthor J. Huard receives remuneration for consulting and royalties from Cook Myosite Inc. The corresponding author K. Weiss is on the scientific advisory board of Eleison Pharmaceuticals. The remaining authors have no conflicts to disclose.
